# Frequency and Prognostic Impact of *CEBPA* Proximal, Distal and Core Promoter Methylation in Normal Karyotype AML: A Study on 623 Cases

**DOI:** 10.1371/journal.pone.0054365

**Published:** 2013-02-01

**Authors:** Annette Fasan, Tamara Alpermann, Claudia Haferlach, Vera Grossmann, Andreas Roller, Alexander Kohlmann, Christiane Eder, Wolfgang Kern, Torsten Haferlach, Susanne Schnittger

**Affiliations:** MLL Munich Leukemia Laboratory, Munich, Germany; University of North Carolina at Chapel Hill, United States of America

## Abstract

The clinical impact of aberrant *CEBPA* promoter methylation (PM) in AML is controversially discussed. The aim of this study was to clarify the significance of aberrant *CEBPA* PM with regard to clinical features in a cohort of 623 cytogenetically normal (CN) *de novo* AML. 555 cases had wild-type *CEBPA,* 68 cases harbored *CEBPA* mutations. The distal promoter was methylated in 238/623 cases (38.2%), the core promoter in 8 of 326 cases (2.5%), whereas proximal PM was never detected. *CEBPA* PM and *CEBPA* mutations were mutually exclusive. *CEBPA* distal PM positive cases were characterized by reduced *CEBPA* mRNA expression levels and elevated white blood cell counts. *CEBPA* distal PM was less frequent in patients with mutations in *FLT3*, *NPM1* and *TET2* and more frequent in cases with *RUNX1* and *IDH2*R140 mutations. Overall, no association of methylation to prognosis was seen. However *CEBPA* distal PM was associated with inferior outcome in cases with low *FLT3*-ITD ratio or *TET2* mutations. A distinct gene expression profile of *CEBPA* distal PM positive cases compared to *CEBPA* mutated and *CEBPA* distal PM negative cases was observed. In conclusion, the presence of aberrant *CEBPA* PM is associated with distinct biological features but impact on outcome is weak.

## Introduction

The CCAAT/enhancer binding protein α (*CEBPA*) is a transcription factor with critical roles in tissue specific gene expression and proliferation arrest. In the hematopoietic system, *CEBPA* expression is restricted to myelomonocytic cells and is specifically up-regulated during granulocyte differentiation [1]. Loss of *CEBPA* function is known to result in a block of granulopoiesis. *CEBPA* has gained interest in the AML field, as it has been shown that down-regulation of *CEBPA* protein through mutations, posttranscriptional modifications and protein-protein interactions with fusion proteins such as *RUNX1-RUNX1T1* or *CBFB-MYH11* plays a key role in leukemic transformation.

Mutations in the *CEBPA* gene have been described for approximately 5–10% of all AML patients and are most common in CN-AML (15%) [2], [3]. In addition to genetic mutations, in recent years, epigenetic modifications, such as DNA promoter hypermethylation have gained increasing interest as additional mechanisms for transcriptional regulation of cancer-related genes. Hence, inactivation of gene expression by abnormal hypermethylation of CpG islands in promoter regions of tumor suppressor genes has been described for many cancer entities [4].

Studies of the *CEBPA* promoter revealed three regions important for promoter function. The core promoter region (−141 to +103 upstream from transcription start site) contains the TATA box and several regulatory factors necessary for *CEBPA* expression [5]. The upstream promoter region (−1422 to −896 upstream from transcription start site) has been shown to interact with MBD2 and MeCP2 methyl-CpG binding proteins and contains binding sites for the transcriptional factors USF−1/−2 and Sp1 suggesting that methylation decreases the cis-activity of these factors, leading to lower *CEBPA* expression. According to methylation levels, the upstream promoter region can be divided into a highly methylated distal region (−1422 to −1121 upstream from transcription start site) and a lowly methylated proximal region (−1121 to −896 upstream from transcription start site) [6].

Recent reports have shown that epigenetic modification of the distal *CEBPA* promoter region resulted in the down regulation of *CEBPA* expression in lung cancer [6], head and neck squamous cell carcinoma [7] and pancreatic cancer cells [8]. Additionally, several studies document epigenetic modification of *CEBPA* in AML. Chim *et al*. [9] found aberrant methylation in the *CEBPA* core promoter (−141 to −15 from transcription start site) in 2/70 unselected AML patients (2.8%). Wouters *et al*. [10] found a correlation between silenced *CEBPA* and frequent *CEBPA* core promoter hypermethylation in six of 285 patients with *de novo* AML (1.4%). Hackanson *et al*. [11] have observed methylation in the distal *CEBPA* promoter region in 20 of 39 (51%) AML patients carrying the recurrent cytogenetic aberrations inv(16), t(8;21), t(15;17), t(9;11) or complex karyotype. Lin et al. [12] evaluated the methylation status of the *CEBPA* core, proximal and distal promoters in a total cohort of 193 unselected patients with *de novo* AML. They found heterogeneous methylation in the distal promoter region, but not in the proximal or core promoter regions. In the total cohort of 193 patients, high *CEBPA* PM was correlated with better treatment response and in a subcohort of 25 CN-AML patients without *CEBPA* and *NPM1* mutations, cases with high *CEBPA* PM had longer overall survival (OS) compared to cases with low *CEBPA* PM. Due to the differences in these studies with respect to selected patient cohorts and the examined *CEBPA* promoter regions, the clinical implications of *CEBPA* methylation in AML remain unclear.

In the present study, we analyzed the methylation status of the *CEBPA* promoter region including core, proximal and distal promoters in 555 *de novo* CN-AML with wt *CEBPA* to clarify the frequency and the significance of aberrant *CEBPA* PM with regard to clinical features. To exclude coincidence of *CEBPA* PM with *CEBPA* mutations, we also analyzed 68 CN-AML cases with *CEBPA* mutations for *CEBPA* PM. In addition, we addressed the question, whether *CEBPA* PM positive AML constitutes a distinct entity in CN-AML. Therefore, we performed global gene expression profiling (GEP) comparing *CEBPA* PM positive cases to *CEBPA* mutated and *CEBPA* unmutated/unmethylated cases.

## Materials and Methods

### Patients

We analyzed a total cohort of 623 de novo AML patients that were referred to our laboratory for first diagnosis of AML between August 2005 and October 2010 (Table 1). AML was diagnosed according to the FAB and WHO classifications [13], [14]. 294 patients were female, 329 male and the median age was 63.9 years (range 20.0–89.6 years). Bone marrow blast percentages ranged from 20 to 99% (median: 67.5%) in 604 patients with non-M6 AML. 19 Patients with AML M6 subtype had blast percentages below 20% (3–17%, median: 12%), as characteristic for the AML M6 subtype. 555 of the 623 cases had CN-AML and wild-type *CEBPA*. Data on other molecular markers was available in: *NPM1*: n = 551, *FLT3*-ITD: n = 552, *FLT3*-TKD: n = 447, *MLL*-PTD: n = 552, *RUNX1*: n = 467, *ASXL1*: n = 420, *IDH1*R132: n = 382, *IDH2*R140: n = 344, and *IDH2*R172: n = 345, *TET2*: n = 113 and *DNMT3A*: n = 119, respectively. For comparison 68 patients with *CEPBA* mutations (38 monoallelic, 20 biallelic, 10 homozygous) were analyzed in addition. Clinical follow up data was available in 435 patients. Patients received standard induction and consolidation chemotherapies such as “7+3”, TAD or HAM. All patients gave written informed consent for scientific studies, e.g. molecular analyses. The study was approved by the Internal Review Board and adhered to the tenets of the Declaration of Helsinki.

**Table 1:** Patient cohorts.

	Cases	Male	Female	Median age, years
	n = 623	n = 329	n = 294	(range)
1. ***CEBPA*** ** wt**	555	293	262	63.9 (20.0–89.6)
2. ***CEBPA*** ** mut**	68	36	32	64.7 (15.7–84.6)
monoallelic	38	22	16	65.1 (22.3–74.3)
biallelic	20	9	11	58.9 (15.7–84.6)
homozygous	10	5	5	62.2 (28.5–78.3)

wt: wild-type; mut: mutated.

### Cytomorphology, Cytogenetics, Immunophenotyping

Cytomorphologic assessment was based on May-Grünwald-Giemsa stains, myeloperoxidase reaction, and non-specific esterase using alpha-naphtyl-acetate as described before and was performed according to the criteria defined in the FAB and the WHO classifications [14]–[16]. Cytomorphology was performed in our laboratory in 526/555 of the *CEBPA*wt cases. In addition, 4 cases were identified as AML by immunophenotyping according to blast cell counts. 25 cases were defined as AML by the sent diagnostic report of the clinical centers. Cytogenetic studies were performed after short-term culture. Karyotypes, analyzed after G-banding, were described according to the International System for Human Cytogenetic nomenclature [17]. Prognostic classification into “favorable”, “intermediate” and “adverse” groups was performed according to the refined MRC classification [18]. Cytogenetic results were available for all patients in the study. Immunophenotyping was performed in our laboratory in 284 cases as described previously [19].

### Isolation and Bisulfite Treatment of Nucleic Acids

DNA was extracted according to a standard procedure from fresh bone marrow or peripheral blood cells after Ficoll separation of mononucleated cells. Bisulfite treatment of genomic DNA was performed using the DNA Methylation Gold Kit (Zymo Research, Orange, CA, USA). Bisulfite treated DNA was used in subsequent DNA methylation analyses which were performed either by methylation-specific polymerase chain reaction (MSP) or bisulfite sequencing.

### Methylation-specific Polymerase Chain Reaction (MSP) and Bisulfite Sequencing

**Figure 1:**
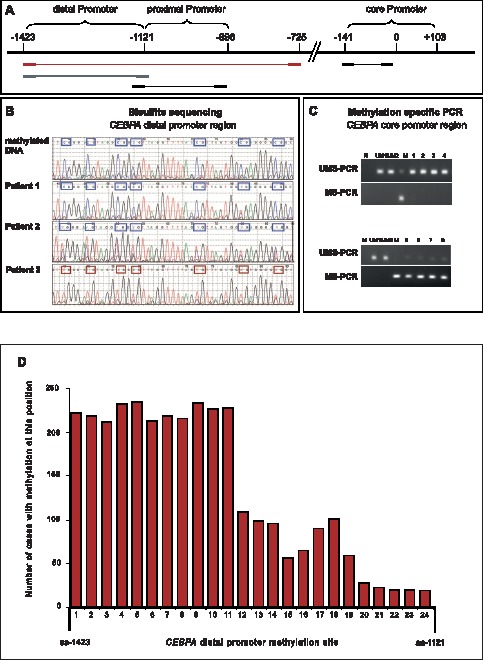
DNA methylation analysis of the CEBPA core promoter and upstream promoter regions. **A)** Scheme of the *CEBPA* promoter region. The areas of DNA methylation analysis are shown below. Red: *CEBPA*-promoter outer primers; grey: *CEBPA* distal PM primers, black: *CEBPA* proximal PM primers; blue: *CEBPA*-U and *CEBPA*-M primers; BS: Bisulfite Sequencing; MSP: Methylation specific PCR. **B)** Bisulfite sequencing results for the distal *CEBPA* promoter region for 3 individual cases compared to positive control. Boxes indicate individual CpG sites analyzed. CG indicates a methylated CpG site; TG indicates an unmethylated CpG site. The distal *CEBPA* promoter region of patient 1 is fully methylated (level of methylation: 75–100%), patient 2 is partly methylated (level of methylation: 20–50%) and patient 3 is unmethylated (level of methylation: <15%). **C)** MSP for *CEBPA* core promoter with *CEBPA*-U and *CEBPA*-M primers. Upper: N: Non-template control; UM1/UM2: positive controls with unmethylated DNA; M: positive control with methylated DNA. 1–4 samples of patients showing positive amplification in UMS-PCR but not in MS-PCR. Lower: 5–8: samples of patients showing positive amplification in MS-PCR but not in UMS-PCR; **D)** Frequency and distribution of methylated CpG islands within the distal *CEBPA* promoter region. Bar chart showing the frequency and distribution of methylated CpG islands within the distal *CEBPA* promoter region for 238 cases with *CEBPA* distal promoter methylation.

Primers used for methylation-specific PCR, bisulfite sequencing and PCR conditions are summarized in Table S1 and were described previously [12], [20]. Locations of individual primers are shown in Figure 1. The PCR products for bisulfite sequencing were purified using the Sephadex® PCR purification system (Sigma-Aldrich, St. Louis, MO, USA), and the methylation patterns were determined using the BigDye Terminator v3.1 Cycle Sequencing kit (Applied Biosystems, Life Technologies, Darmstadt, Germany) on an automated ABI 3730 Genetic Analyzer (Applied Biosystems, Life Technologies, Darmstadt, Germany). Bisulfite treated CpGenome universal methylated DNA (Chemicon, Temecula, CA, USA) was used as a methylation-positive control. We analyzed 24 individual CpG dinucleotides in the distal promoter region (−1423 to −1121 upstream from transcription start site) and 20 individual CpG dinucleotides in the proximal promoter region (−1121 to −896 upstream from transcription start site). To evaluate methylation ratio, we counted the amount of cytosines with methylation and the level of methylation for each cytosine by Sanger Sequencing analysis [21]. The percentage of methylation was calculated as the peak height of cytosine vs the peak height of thymine for each CpG site. A single cytosine at the corresponding CpG site was considered as 100% methylation, a single thymine as no methylation and overlapping cytosine and thymine as partial methylation (15–100%). Cytosines were counted as methylated if the methylation intensity was 15% or higher. (Figure 1B/Table S2). Because the PCR products were not cloned, this analysis represents an approximation of the “average” methylation status at each CpG residue for each patient.

### RNA Isolation, Reverse Transcription and Quantitative Real-time PCR

Isolation of mononucleated cells, mRNA extraction, and random primed cDNA synthesis was performed as described previously [22]. RQ-PCR was performed by the use of the Applied Biosystems 7500 Fast Real Time PCR System with the application of specific CEBPA TaqMan Gene Expression Assay (Assay ID: HS00269972_S1). Amplification was performed after initial incubation at 95°C for 1 minute in a 3-step cycle procedure (denaturation 95°C, 20 seconds, ramp rate 20°C/s, annealing temperature 60°C, 45 seconds, ramp rate 20°C/s, and extension 72°C, 26 seconds, ramp rate 2°C/s) for 50 cycles. The expression of *CEBPA* was normalized against the expression of the control gene *ABL1* to adjust for variations in mRNA quality and efficiencies of cDNA synthesis. The *CEBPA* expression levels are given as: %*CEBPA/ABL1*.

### Global Gene Expression Profiling

To detect underlying common differences in their gene expression profiles (GEP) we investigated 9 *CEBPA* methylated (unmutated), 8 *CEBPA* single-mutated, 10 *CEBPA* double-mutated, and 10 non-methylated/non-mutated cases (Affymetrix HG-U133 Plus 2.0 microarrays; Santa Clara, CA). All cases analyzed were taken from the subcohorts described above. The microarray sample preparation assay was performed as previously reported [23]. Gene expression raw data was processed according to the manufacturer’s recommendations. After quality control, raw data was normalized for visualization and interpretation using the Robust Multichip Average *(*RMA*)* algorithm as implemented in the R-package *affy* version 1.18.0. Detection calls, i.e. present, marginal, or absent expression, were determined by default parameters. Probe sets were filtered out by the genefilter package. Probe intensities were considered if the normalized signal was above 100 (unlogged data). Significantly regulated genes were determined using the LIMMA toolbox. A gene was determined as significantly regulated if the p-value was <0.05 after multiple testing correction by Benjamini-Hochberg procedure [24]. Clustering of expression data was performed using Manhattan distance function and complete clustering. To find significant associated biological processes, we performed a Gene Ontology term enrichment analysis, which was carried out with the R package GOstats [PMID: 15461798]. Raw data is available at GEO with accession number GSE34733.

### Statistical Analyses

Survival curves were calculated for overall survival (OS), event free survival (EFS) and relapse free survival (RFS) according to Kaplan-Meier and compared using the two-sided log rank test. OS was the time from diagnosis to death or last follow-up. EFS was the time from diagnosis to treatment failure, relapse, death, or last follow-up in complete remission. RFS was the time from achievement of complete remission to relapse, death, or last follow-up in complete remission. Complete remission and relapse were defined according to Cheson et al. [25]. Cox regression analysis was performed for OS and EFS with different parameters as covariates. Median follow-up was calculated taking into account the respective last observations in surviving cases and censoring non-surviving cases at the time of death. Parameters which were significant in univariable analyses were included into multivariable analyses. Dichotomous variables were compared between different groups using the χ^2^-test and continuous variables by Student’s T-test and Spearman’s rank correlation. Results were considered significant at p≤0.05. All reported p-values are two-sided. No adjustments for multiple comparisons were performed. SPSS version 19.0 (IBM Corporation, Armonk, NY) was used for statistical analysis.

## Results

### Distribution of *CEBPA* Promoter Methylation

We evaluated the *CEBPA* promoter methylation status in a total cohort of 623 patients with *de novo* CN-AML using methylation-specific PCR and bisulfite sequencing methods. This cohort comprised of 555 cases with *CEBPA* wild-type (wt) and 68 *CEBPA* mutated (mut) status.

Methylation specific PCR analysis revealed *CEBPA* core PM in only 8 of the first 326 cases analyzed (2.5%) (Figure 1C/Table 2). Because of this low frequency we did not continue with this analysis. The total cohort of 623 cases was subsequently analysed using semiquantitative bisulfite sequencing. In the cohort of 555 CN-AML cases with *CEBPA*wt we identified 238 of 555 cases (42.9%) with methylated CpG sites in the distal promoter region (*CEBPA* dPM^pos^) (Table S2). The amount of methylated cytosines ranged from 2 to 24 and the methylation levels ranged from 15% to 100% compared to positive control (Figure 1 B). The cytosines of the first 11 CpG sites of the distal *CEBPA* promoter were more often methylated than the 13 cytosines of the C-terminal CpG sites (Figure 1D). We next defined a threshold among the 238 *CEBPA* dPM^pos^ cases by forming a ratio of the methylation intensity and the amount of methylated cytosines (see Materials and Methods). Mean methylation ratio was 541. Cases with a ratio less than 541 were defined as lowly methylated (*CEBPA* dPM^low^), cases with a ratio higher than 541 as highly methylated (*CEBPA* dPM^high^). According to this, 144/238 (60.5%) cases were *CEBPA* dPM^low^ and 94/238 (39.5%) cases were *CEBPA* dPM^high^. Methylation of the 20 individual CpG dinucleotides in the proximal promoter was not detected (Table 2).

**Table 2:** Frequency and distribution of *CEBPA* promoter methylation.

	Cases	*CEBPA*	*CEBPA*	*CEBPA*
		Core	Proximal	Distal
	n =	Promoter	Promoter	Promoter
***CEBPA*** ** wt**	**555**	8/326 (2.5%)	0/572	238/555 (42.9%)
***CEBPA*** ** mut**	**68**			
monoallelic	38	0	0	0
Biallelic	20	0	0	0
homozygous	10	0	0	0

wt: wild-type; mut: mutated.

One single patient with AML M0 subtype carried methylation throughout both the distal and the core *CEBPA* promoter.

None of the 68 *CEBPA* mutated cases harbored methylation in any promoter region analyzed and thus aberrant *CEBPA* PM and mutation status were mutually exclusive (Table 2).

### Cytomorphology and Immunophenotyping

According to the FAB classification system, of the 526/555 *CEBPA*wt cases with cytomorpholocical data 28 were AML M0, 162 AML M1, 183 AML M2, 114 AML M4, 13 AML M5, 17 AML M6 and one AML M7 in FAB subgroups, respectively. In 8 cases FAB classification was not possible. 284 cases were analyzed by immunophenotyping in addition. Cases with *CEBPA* distal PM as compared to those without revealed a more immature phenotype with stronger expression of CD34 (mean positive cells 33±29% vs. 27±26%, p = 0.038) and CD133 (mean positive cells 26±29% vs. 17±23%, p = 0.030) and a weaker expression of CD64 (mean positive cells 33±24% vs. 43±27%, p = 0.002). Furthermore, we observed that the T-lymphoid marker CD7 was significantly stronger expressed in cases with *CEBPA* distal PM (mean±SD positive cells 29±23% vs. 20±19%; p = 0.001).

### Influence of *CEBPA* Distal PM on *CEBPA* Expression

**Figure 2:**
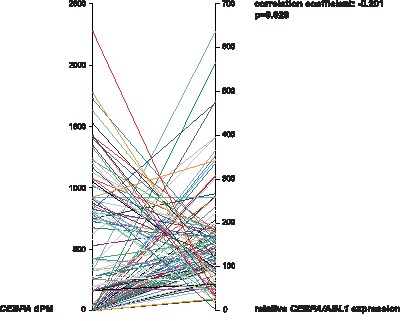
*CEBPA* expression correlates with promoter methylation levels. Spearman’s rank correlation of *CEBPA* expression levels to *CEBPA* methylation levels shows a significant inverse correlation (Spearman correlation coefficient = -0,201, p = 0.023).

To determine whether aberrant DNA methylation in the distal promoter affects *CEBPA* expression, we analyzed *CEBPA* expression levels in 120/555 cases by quantitative real-time RT-PCR. Median *CEBPA* expression level was 134.7 (range: 2.7–637.0). We correlated *CEBPA* expression levels to *CEBPA* methylation levels by Spearman’s rank correlation and found a limited but significant inverse correlation (Spearman correlation coefficient = −0.201, p = 0.023; Figure 2). We conclude that the DNA methylation in the *CEBPA* distal promoter region correlates at least in part with the downregulation of *CEBPA* expression in CN-AML patients and that also other causes for DNA methylation must be considered.

### Correlation to Clinical Features

There was no significant difference in median age between the *CEBPA* dPM^pos^ compared to *CEBPA* dPM negative (*CEBPA* dPM^neg^) cases (64.3 vs. 64.4 years). The same holds true for platelet count (median 97.7 vs. 95.8×10^9^/L, n.s.) and hemoglobin level (median 9.6 vs. 9.2×g/dL, n.s.) (Table 3). Bone marrow blast percentage also did not differ between *CEBPA* dPM^pos^ and *CEBPA* dPM^neg^ cases (median 58.0% vs. 68.5%; n.s.). Furthermore, there was no correlation between bone marrow blast percentage and *CEBPA* dPM threshold (data not shown). Solely, the white blood cell count (WBC) was significantly lower in *CEBPA* dPM^pos^ compared to *CEBPA* dPM^neg^ cases (median 34.6 vs. 50.9×10^9^/L, p = 0.003). To analyze whether the *CEBPA* dPM threshold is decisive for an elevated WBC count, we compared *CEBPA* dPM^low^ to *CEBPA* dPM^high^ cases. There was no significant difference in the WBC counts in these cases (median 36.6 vs. 31.3×10^9^/L; n.s.), indicating that the *CEBPA* dPM status per se and not *CEBPA* dPM ratio impacts on the WBC count.

**Table 3:** Patient characteristics of *CEBPA* distal promoter methylation positive cases.

	Total n = 555	*CEBPA* distal PM negative, n = 317	*CEBPA* distal PM positive, n = 238	p =
**Sex**				
Female	262 (47.2%)	151/262 (57.6%)	111/262 (42.3%)	n.s.
Male	293 (52.8%)	165/293 (56.3%)	128/293 (43.7%)	n.s.
**Age [years] median (range)**	63.9 (20.0–89.6)	64.4 (20.9–89.6)	63.3 (20.0–87.6)	n.s.
**BM blasts, % median (range)**	65 (3–99)	68.5 (4–99)	58 (3–99)	n.s.
**WBC count [×10^9^/L] median (range)**	43.9 (0.6–400.0)	50.9 (0.6–400.0)	34.6 (0.7–187.1)	0.003
**Hb level [g/dL] median (range)**	9.3 (2.8–16.3)	9.2 (2.8–16.3)	9.6 (5.0–14.2)	n.s.
**Platelet count [×10^9^/L] median (range)**	96.6 (3.0–950.0)	95.8 (3.0–950.0)	97.7 (17.0–363.0)	n.s.

n.s.: not significant; BM: bone marrow; WBC: white blood cell; Hb: hemoglobin.

*CEBPA* core PM positive cases showed no differences in age, WBC count, platelet count and hemoglobin levels compared to *CEBPA* core PM negative cases (data not shown). Due to the limited number of cases, we did not perform further analysis on *CEBPA* core PM positive cases.

### Association with Other Mutations

To determine, whether *CEBPA* dPM correlates with mutations frequently reported in AML we analyzed *FLT3*-ITD and *MLL*-PTD as well as mutations in *NPM1*, *FLT3*-TKD, *RUNX1*, *ASXL1*, *DNMT3A*, *IDH1*R132, *IDH2*R140, *IDH2*R172 and *TET2* in correlation to *CEBPA* dPM status. Cases positive for *FLT3*-ITD with ratio <0.5 were grouped together with the *FLT3*-ITD negative cases, as it has been shown that only a *FLT3*-ITD ratio >0.5 has a significant adverse prognostic impact [26]. Thus, *FLT3*-ITD negative patients and patients with *FLT3*-ITD ratio <0.5 are combined and designated as *FLT3*-ITD/*FLT3*wt^ratio<0.5^. *NPM1* mutations (90/237, 38.0% vs. 160/314, 51.0%, p = 0.003), *FLT3-*ITD*/FLT3*wt^ratio<0.5^ (32/238, 13.4% vs. 66/314, 21.0%; p = 0.024) and *TET2* mutations (10/58, 17.2% vs. 21/55, 38.2%; p = 0.02) were less frequent in the *CEBPA* dPM^pos^ compared to *CEBPA* dPM^neg^ cases while *IDH2*R140 mutations (41/153, 26.8% vs. 28/191, 14.7%; p = 0.007) were significantly more frequent. *NPM1* mutated cases and *TET2* mutated cases showed significantly lower *CEBPA* dPM ratios compared to the respective wt cases while cases with a *FLT3*-ITD ratio <0.5 and those with *IDH2*R140 mutations showed significantly higher *CEBPA* dPM ratios as compared to the respective control cases. Furthermore, we observed a positive correlation of *CEBPA* dPM ratio with *RUNX1* mutations (Table 4). Moreover, we analyzed, whether a *CEBPA* dPM threshold is important for the correlation to the above described mutations. Comparison of *CEBPA* dPM^high^ versus *CEBPA* dPM^low^ cases revealed that solely the frequency of *NPM1* mutations was significantly higher in *CEBPA* dPM^low^ compared to *CEBPA* dPM^high^ cases (62/143, 43.4% vs. 28/94, 29.8%; p = 0.04). In contrast, there was no correlation of *CEBPA* dPM threshold to *FLT3*-ITD ratio, *TET2*, *RUNX1* or *IDH2*R140 mutations (data not shown).

**Table 4:** Correlation of *CEBPA* distal promoter methylation ratio to molecular mutations.

Mutation (n = cases analyzed)	*CEBPA* distal PM ratio ± s.d.	p-value
***NPM1*** ** (n = 551)**		
wt (n = 301)	295.4±434.9	<0.001
mut (n = 250)	158.2±295.2	
***FLT3*** **-ITD (n = 552)**		
Negative and ratio<0.5 (n = 454)	248.6±393.4	0.026
ratio>0.5 (n = 98)	163.8±326.8	
***FLT3*** **-TKD (n = 447)**		
wt (n = 399)	237.1±397.4	n.s.
mut (n = 48)	268.3±364.7	
***MLL*** **-PTD (n = 552)**		
neg (n = 477)	226.7±384.1	n.s.
pos (n = 75)	271.7±380.6	
***RUNX1*** ** (n = 467)**		
wt (n = 356)	216.8±366.9	0.049
mut (n = 111)	308.7±444.1	
***ASXL1*** ** (n = 420)**		
wt (n = 353)	226.3±388.3	n.s.
mut (n = 67)	270.7±416.6	
***TET2*** ** (n = 113)**		
wt (n = 82)	290.2±380.1	0.034
mut (n = 31)	131.9±252.5	
***IDH1*** **R132 (n = 382)**		
wt (n = 347)	229.9±394.6	n.s.
mut (n = 35)	216.1±341.6	
***IDH2*** **R140 (n = 344)**		
wt (n = 275)	213.2±377.5	0.025
mut (n = 69)	354.9±480.3	
***IDH2*** **R172 (n = 345)**		
wt (n = 335)	230.8±388.6	n.s.
mut (n = 10)	270.0±357.4	
***DNMT3A*** ** (n = 119)**		
wt (n = 74)	352.3±499.0	<0.001
mut (n = 45)	156.3±290.4	

wt: wild-type; mut: mutated.

### Prognostic Relevance of *CEBPA* Promoter Methylation

**Figure 3:**
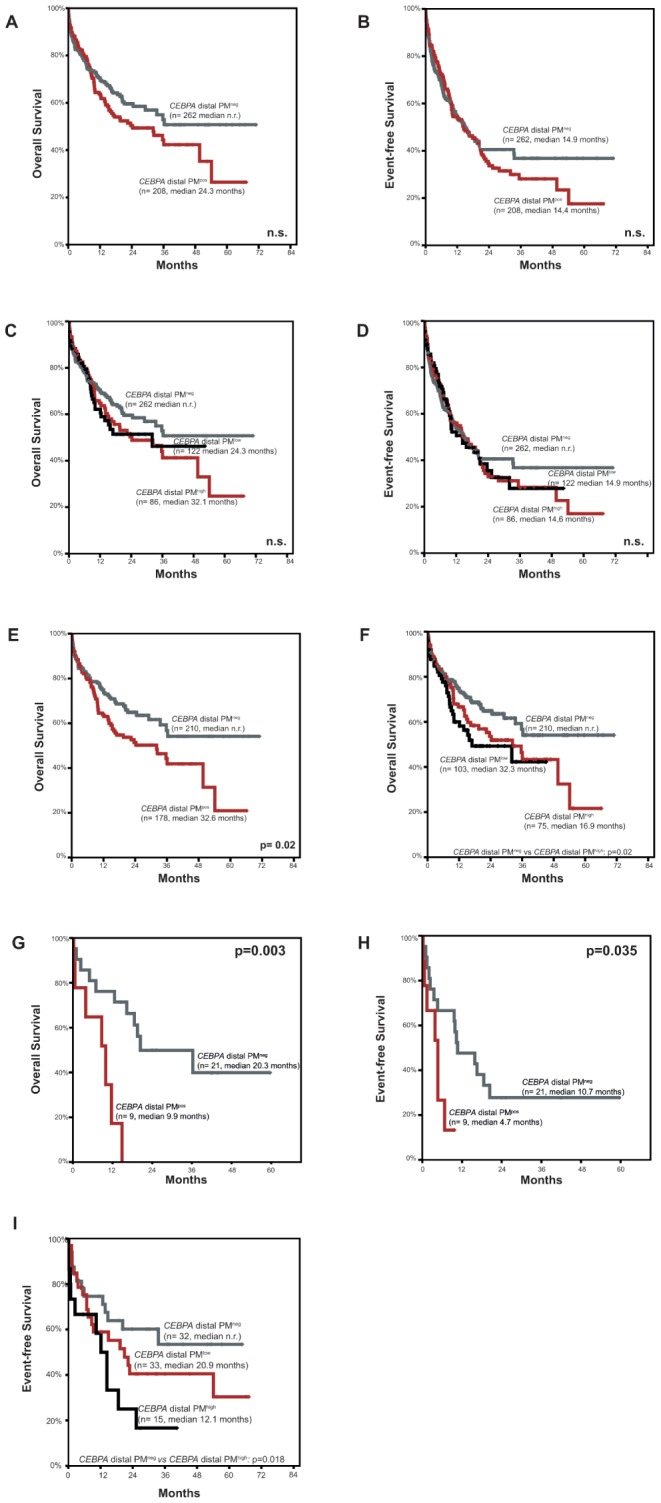
Kaplan Meier survival analysis according to *CEBPA* distal promoter methylation status. Survival within the total cohort of 470 patients with CN-AML and *CEBPA*wt. Kaplan Meier plot showing **A)** Overall and **B)** Event-free survival of *CEBPA* distal promoter methylation positive (red) compared to *CEBPA* distal promoter methylation negative cases (grey). **C)** and **D)** Overall survival and event-free survival within the total cohort of 470 patients according to *CEBPA* distal PM threshold. Kaplan Meier plot of *CEBPA* distal promoter methylation high cases (red) compared to *CEBPA* distal promoter methylation low (black) and *CEBPA* distal promoter methylation negative cases (dark grey). **E)** Survival Analysis within the cohort of 388 cases with CN-AML with *FLT3*-ITD ratio <0.5. Kaplan Meier plot showing overall survival according to *CEBPA* distal promoter methylation status of *CEBPA* distal promoter methylation positive (red) compared to *CEBPA* distal promoter methylation negative cases (grey). **F)** Overall survival within the cohort of 388 cases with CN-AML with *FLT3*-ITD ratio <0.5.according to *CEBPA* distal promoter methylation threshold of *CEBPA* distal promoter methylation high cases (red) compared to *CEBPA* distal promoter methylation low (black) and *CEBPA* distal promoter methylation negative cases (dark grey). **G)** Survival analysis according to *CEBPA* distal promoter methylation status within the cohort of 30 cases with CN-AML and *TET2* mutations. Kaplan Meier plot showing overall survival and **H)** Event-free survival of *CEBPA* distal promoter methylation positive (red) compared to *CEBPA* distal promoter methylation negative cases (grey). **I)** Event-free survival within the cohort of 80 patients with CN-AML and *TET2* wild-type according to *CEBPA* distal promoter methylation threshold. Kaplan Meier plot of *CEBPA* distal promoter methylation high cases (black) compared to *CEBPA* distal PM low (red) and *CEBPA* distal promoter methylation negative cases (dark grey).

First, we analyzed, whether *CEBPA* dPM status has impact on prognosis. *CEBPA* dPM^pos^ cases did not significantly differ from *CEBPA* distal PM^neg^ cases with regard to OS (24.3 months vs. median n.r.; n.s.) and EFS (median 14.4 months vs. 14.9 months; n.s.). (Figure 3 A+B). Applying the *CEBPA* dPM threshold also revealed no significant impact of *CEBPA* dPM^high^ versus *CEBPA* dPM^low^ and *CEBPA* dPM^neg^ status on OS (median 32.1 months vs. 24.3 months vs. median n.r. respectively; n.s.) and EFS (median 14.4 months vs. 14.9 months vs. 14.9 months, respectively; n.s.) (Figure 3 C+D). As for the observed association of *CEBPA* dPM with other molecular markers, we performed further subcohort analysis. No prognostic impact of *CEBPA* dPM was seen in subcohorts defined by age, *NPM1* mutations or *IDH*R140 mutations (data not shown). However, OS in patients with *FLT3*wt/*FLT3*-ITD^ratio<0.5^ was significantly worse in cases with additional *CEBPA* dPM compared to those without (32.6 months vs. median n.r., p = 0.02). Moreover, the threshold of *CEBPA* dPM seemed to be of importance for patients with *FLT3*wt/*FLT3*-ITD^ratio<0.5^, as *CEBPA* dPM^high^ cases showed significantly worse OS compared to *CEBPA* dPM^neg^ cases (16.9 months vs. median n.r.; p = 0.03) (Figure 3 E+F). Furthermore, patients with *TET2*mut and *CEBPA* dPM^pos^ had significantly worse OS (median 9.9 months vs. 20.3 months, p = 0.003) and EFS (median 4.7 months vs. 10.7 months; p = 0.035) compared to those *TET2*mut patients with *CEBPA* dPM^neg^ (Figure 3 G+H). With regard to *CEBPA* dPM threshold, survival analysis of *TET2* mutated cases was not valid, as patient numbers were too small. In the *TET2*wt subcohort however, we observed significantly worse EFS for *CEBPA* dPM^high^ compared to *CEBPA* dPM^neg^ cases (12.1 months vs. median n.r.; p = 0.018) (Figure 3 I). We also analyzed outcome according to *CEBPA* methylation status compared to *CEBPA* mutation status and found no significant impact for the methylation status (Figure S1).

### Uni- and Multivariable Analysis

In univariable analysis, the following parameters were associated with worse EFS: higher age (p<0.001), higher WBC count (p<0.001), *FLT3*-ITD/wt ratio higher than 0.5 (p<0.001), and *RUNX1* mutations (p = 0.003). *NPM1* mutations (p = 0.011) were associated with better EFS. *CEBPA* dPM status or *CEBPA* dPM ratio had no significant influence on EFS. In multivariable analysis, only age (P<0.001), WBC count (p<0.001) and *FLT3*-ITD/wt ratio <0.5 (p = 0.001) maintained their relevance for EFS. Investigating OS, age (p<0.001) and WBC (p<0.001), the *FLT3*-ITD ratio >0.5 (P = 0.014), *NPM1* mutations (p<0.001) and *RUNX1* mutations (p = 0.001) were prognostically relevant in univariable analysis. *CEBPA* dPM status or *CEBPA* dPM ratio had no significant impact on OS in univariable analysis. In multivariable analysis, age (p<0.001), WBC count (p<0.001), *FLT3*-ITD/wt ratio >0.5 (p = 0.012) and *NPM1* mutations (p = 0.007) retained their prognostic impact (Table 5).

**Table 5:** Influence of different biological and leukemia-associated parameters on OS and EFS in 555 CN-AML patients in uni- and multivariable analysis.

Parameter	EFS univariable		EFS multivariable		OS univariable		OS multivariable	
	P	RR	P	RR	P	RR	P	RR
Age	<0.001	1.34*	<0.001	1.41*	<0.001	1.49*	<0.001	1.54*
Gender	NS	–	–	–	NS	–	–	–
WBC count	<0.001	1.06#	<0.001	1.06#	<0.001	1.06#	<0.001	1.08#
CEBPA dPM status	NS	–	–	–	NS	–	–	–
CEBPA dPM ratio	NS	–	–	–	NS	–	–	–
FLT3-ITD/wt ratio (≥0.5)	<0.001	1.74	0.001	1.91	0.014	1.55	0.012	1.93
NPM1mut	0.011	0.72	NS	0.79	<0.001	0.52	0.007	0.51
RUNX1mut	0.003	1.56	NS	–	0.001	1.75	NS	–

Abbreviations: EFS:event-free survival; NS: not significant; OS: overall survival; RR: relative risk.

* Per 10 years of increase;

# Per 10×10^9^/L.

Age, peripheral blood cell counts and *CEBPA* dPM ratio were considered as continuous parameters.

### Global Gene Expression Profiling

We compared the gene expression profiles of 9 *CEBPA* dPM samples with 8 *CEBPA* single-mutated, 10 *CEBPA* double-mutated and 10 *CEBPA* non-methylated/non-mutated cases. For the multicomparison, significant differential expression was detected for a total of 727 genes.

We identified 548 genes for the comparison of *CEBPA* methylated and non-methylated/non-mutated samples. Comparison of *CEBPA* methylated with double-mutated samples revealed 298 significantly differentially expressed genes. Analyzing these pairwise comparisons, we identified overlapping 119 genes, which were significantly differentially expressed for both sets (Tables S4/S5). We identified no significantly differentially expressed genes for *CEBPA* methylated and single-mutated samples, as *CEBPA* single-mutated cases showed a strong heterogeneous expression pattern (compare Figure S2).

To further study the expression profiles we then applied clustering algorithms. *CEBPA* methylated samples showed a unique pattern compared to the remaining samples (*CEBPA* single-mutated, *CEBPA* double-mutated and *CEBPA* non-methylated/non-mutated cases). By Gene Ontology analysis, significantly differentially expressed genes for the multicomparison were associated with a function for myeloid cell differentiation and hematopoietic development, e.g. *RUNX1* was upregulated and Kruppel-like factor 1 (*KLF1*) was downregulated (Table S3).

## Discussion

In the present study, we investigated the frequency and the clinical relevance of *CEBPA* PM in 623 *de novo* CN-AML and showed that aberrant DNA methylation in the promoter of *CEBPA* is very heterogeneously spread across the core, proximal and distal promoter regions. A distinct pattern of aberrant DNA methylation was mainly restricted to the distal promoter region of *CEBPA* (42.9%), whereas methylation of the *CEBPA* core promoter seems to be a rare event in AML (2.5%). Methylation of the *CEBPA* proximal promoter was not observed in any case. These findings are in line with previous reports [11], [12], [27]. Coincidence of *CEBPA* PM and *CEBPA* mutations was never observed, indicating that these two events are mutually exclusive.

Aberrant *CEBPA* PM has also been described in lung cancers and head and neck cancers. The core promoter was not affected by epigenetic silencing in these entities [6], [7]. It is noteworthy that the DNA methylation patterns within the CpG islands showed tumor-type specificity with *CEBPA* methylation being restricted to the distal promoter region in head and neck cancer [24], whereas in lung cancer also the proximal promoter region was differentially methylated [23]. In contrast, in AML *CEBPA* methylation could be observed in the distal promoter region as well as in the core promoter region. A possible explanation for this finding could be that different regulatory regions are used in different tissues, and epigenetic mechanisms interrupt the interaction of the relevant binding proteins with these regions through chromatin conformation changes.

Data regarding influence of *CEBPA* PM on *CEBPA* expression in AML is heterogeneous (an overview is given in Table S6). This is probably due to the variability in the selected cohorts as well as the *CEBPA* promoter region analyzed. Hollink et al [27] analyzed the relevance of *CEBPA* core PM in 237 unselected cases of pediatric AML and found it to be a rare event, as it occurred in only three cases (1.3%). Furthermore, *CEBPA* gene expression was down regulated in these cases. Wouters et al. [10] showed in a cohort of 285 unselected AML patients that *CEBPA* silencing is not associated with *CEBPA* hypermethylation, suggesting a possible yet unknown mechanism of *CEBPA* mRNA repression. Lin et al. [12] correlated the methylation levels in the distal *CEBPA* promoter region with its transcript levels in leukemic cells prepared from 12 unselected AML patients and observed a negative correlation. They conclude that the DNA methylation in the distal *CEBPA* promoter region correlates with the down regulation of *CEBPA* expression in patients with AML. We were able to confirm this data, as we also found *CEBPA* expression to be negatively correlated with *CEBPA* distal PM in CN-AML cases in the present study.

Another interesting finding was the aberrant expression of the T-cell marker CD7 of CEBPA dPM positive cases, which has already been reported by Wouters et al. [10]. In contrast, we were not able to confirm a mixed myeloid/T-lymphoid phenotype, as we did not detect an increased expression of myeloid markers like CD13 and CD33.

To date, there is only one study by Lin et al. regarding prognostic relevance of *CEBPA* distal PM in AML [12]. This report describes favorable prognosis for AML patients with *CEBPA* PM in a subcohort of 59 cases after excluding patients with favorable karyotypes, *NPM1* mutations and *CEBPA* mutations. Furthermore a survival advantage for patients with *CEBPA* promoter hypermethylation was seen within a subcohort of 25 CN-AML patients with wt *CEBPA* and wt *NPM1*. However, these results were based on a relatively small number of cases analyzed. Furthermore, in multivariable analysis, they found high *CEBPA* PM to be an independent prognostic factor for disease-free survival. In another paper Hollink et al. [27] performed unsupervised cluster analysis in 237 unselected cases of pediatric AML and identified five cases with silenced *CEBPA*, including three cases with aberrant *CEBPA* PM. Four of these cases experienced relapse indicating poor outcome for patients with silenced *CEBPA*.

With regard to prognosis, our survival data of 470 patients show for the first time that *CEBPA* distal PM per se is not a prognostic factor in the overall cohort of CN-AML. Furthermore, the survival analysis of a subcohort of 260 CN-AML patients with wt *NPM1* and wt *CEBPA* revealed no prognostic impact of *CEBPA* distal PM (data not shown) and thus is in contrast to the study of Lin et al. [12]. However, we observed an adverse impact of high *CEBPA* distal PM on OS in the subset of cases with *FLT3*wt/*FLT3*-ITD^ratio<0.5^. But not only in this more favorable group a prognostic effect could be shown. Also in the more adverse subgroup with *TET2* mutations the *CEBPA* dPM^pos^ had significantly worse OS and EFS compared to those with *TET2* mutations and *CEBPA* dPM^neg^. However, this result is based on only a limited number of patients and needs to be validated in a larger cohort. In multivariable analysis, *CEBPA* dPM had no significant impact on OS and EFS in our series, which is again in contrast to the study of Lin et al.

Taken together, our data is in line with the concept that *CEBPA* PM does not directly influence prognosis. We rather assume that the negative prognostic effect of *CEBPA* PM which was observed in certain subgroups is caused by the *CEBPA* PM induced down regulation of *CEBPA* expression. This concept is supported by the study of Figueroa et al. [28] which showed that *CEBPA* silenced cases (n = 8) had a considerably worse outcome compared to *CEBPA* mutated cases (n = 8) (5-year overall survival 25% vs. 88%; log-rank test *P*<0.003). Furthermore, Barjesteh et al. were also able to show an unfavorable prognosis for six patients with intermediate-risk karyotype AML and low *CEBPA* expression [29].

Data on gene expression profiles of *CEBPA* methylated AML are heterogeneous. In the study of Hollink et al [27], unsupervised cluster analysis of the total cohort of 237 cases showed that *CEBPA* mutated cases predominantly clustered together with *CEBPA* hypermethylated cases. Figueroa et al. [28] performed unsupervised cluster analysis and also found a similar gene expression profile of *CEBPA* silenced AML and *CEBPA* mutated AML. In contrast to these studies, our gene expression analysis of *CEBPA* PM^pos^ cases showed a highly distinct clustering of *CEBPA* methylated cases compared to *CEBPA* mutated cases, emphasizing the relevance of the aberrant *CEBPA* distal PM. Our data is affirmed by our recently published study on gene expression profiling in AML [30]. In this study gene expression signatures for 30 *CEBPA* mutated cases were compared with the profiles of 204 *CEBPA* wt cases. *CEBPA* mutated cases and *CEBPA* wt cases showed a highly distinct gene expression signature and did not cluster together. As in the present study all *CEBPA* distal PM positive cases are *CEBPA* unmutated, it is feasible that they show a distinct gene expression profile from *CEBPA* mutated cases.

It can be speculated that CEBPA promoter methylation is not a focal, targeted event and that it may perhaps more likely occur in the context of global hypermethylation as observed by Figueroa [28]. Such analysis should be in the focus of further evaluation.

In conclusion, we demonstrate that aberrant methylation of the distal *CEBPA* promoter can be found in a substantial proportion of CN-AML patients. It is positively correlated to genotypes with *RUNX1*mut and *IDH2*R140mut and negatively correlated with *NPM1*mut, *FLT3*-ITD, *TET2*mut and *DNMT3A*mut. An effect of epigenetic modifications of the *CEBPA* promoter on survival was not found in the total cohort of 555 CN-AML patients. However, we detected an adverse effect in the subsets with *FLT3*wt/*FLT3*-ITD^ratio<0.5^ and those with *TET2*mut but only in univariable analysis. Furthermore, aberrant methylation of the distal *CEBPA* promoter was closely correlated to reduced *CEBPA* expression and a distinct gene expression profile. This change in underlying gene expression profile suggests a contribution of *CEBPA* methylation to leukemic transformation. However, *CEBPA* distal PM is a negligible prognostic marker, as we show that its influence on outcome in CN-AML is strongly dependent on other markers.

## Supporting Information

Figure S1 **Gene expression profiling.** Heatmap visualizing the gene expression profiles of 9 *CEBPA* methylated/unmutated 10 *CEBPA* double-mutated, 8 *CEBPA* single-mutated and 10 non-methylated/unmutated cases. The black symbols indicate the *CEBPA* methylated cases, the red symbols *CEBPA* double-mutated cases, the blue symbols *CEBPA* single-mutated cases and the grey symbols the *CEBPA* non-methylated/wild-type cases.
(EPS)

Figure S2 **Kaplan Meier survival analysis according to** ***CEBPA*** **mutation- and distal promoter methylation status.** Survival within the total cohort of 555 patients with CN-AML. Kaplan Meier plot showing A) Overall and B) Event-free survival of *CEBPA*bi mutated (grey) and *CEBPA*mono+homo mutated cases (red) compared to *CEBPA* distal promoter methylation high cases (black) compared to *CEBPA* distal promoter methylation low (purple) and *CEBPA* distal promoter methylation negative cases (blue).
(EPS)

Table S1 **Primers and PCR conditions.**
(DOC)

Table S2 **Methylation status at each CpG residue for each patient.**
(XLS)

Table S3 **Significantly expressed genes with function in regulation of cellular and component organization identified by Gene Ontology.**
(DOC)

Table S4 **Significantly expressed genes with a function for myeloid cell differentiation and hemopoietic development identified by Gene Ontology.**
(DOC)

Table S5 **Significantly expressed genes in Gene Ontology.**
(DOC)

Table S6 **Overview of** ***CEBPA*** **promoter methylation studies.**
(DOC)
