# Supplementary material for: Frequency and Prognostic Impact of CEBPA Proximal, Distal and Core Promoter Methylation in Normal Karyotype AML: A Study on 623 Cases
Source: PLoS One. 2013 Feb 1;8(2):e54365. doi: 10.1371/journal.pone.0054365 (PMC3562230; doi:10.1371/journal.pone.0054365)
Supplement: Table S1 — Primers and PCR conditions. (DOC) [file pone.0054365.s003.doc]

Table S1: Primers and PCR conditions

| **Primer** | **Sequence (5’-3’)** | **annealing temp °C** | **Product size (bp)** |
| --- | --- | --- | --- |
| **Core Promoter Methylation** |  |  |  |
| *CEBPA* U-F | 5’-TTGTTGGGTATAAAAGTTGGGTTGGT-3’ | 60 | 128 |
| *CEBPA* U-R | 5’-AAAATTCTCCCAACATAACAAACCTCA-3’ | 60 | 128 |
| *CEBPA* M-F | 5’-GTCGGGTATAAAAGTTGGGTCGGC-3’ | 65 | 128 |
| *CEBPA* M-R | 5’-ATTCTCCCGACATAACGAACCTCG-3’ | 65 | 128 |
| **Proximal & Distal Promoter Methylation** |  |  |  |
| *CEBPA* promoter outer-F | 5’-TTGTTAGGTTTAAGGTTATTG-3’ | 58 | 320 |
| *CEBPA* promoter outer-R | 5’-CTTTCTTTATAATTACTTCTCCA-3’ | 58 | 320 |
| *CEBPA* proximal PM-F | 5’-TATTTAAGGGGTTTTAGG-3’ | 58 | 280 |
| *CEBPA* proximal PM-R | 5’-AAAAACAAACTTAACTCTAAA-3’ | 58 | 280 |
| *CEBPA* distal PM-F | 5’-TTGTTAGGTTTAAGGTTATTG-3’ | 58 | 310 |
| *CEBPA* distal PM-R | 5’-AAACCCTAAAACCCCTTA-3’ | 58 | 310 |

U: unmethylated; M: methylated; F: forward; R: reverse
