# Supplementary material for: Frequency and Prognostic Impact of CEBPA Proximal, Distal and Core Promoter Methylation in Normal Karyotype AML: A Study on 623 Cases
Source: PLoS One. 2013 Feb 1;8(2):e54365. doi: 10.1371/journal.pone.0054365 (PMC3562230; doi:10.1371/journal.pone.0054365)
Supplement: Table S3 — Significantly expressed genes with function in regulation of cellular and component organization identified by Gene Ontology. (DOC) [file pone.0054365.s005.doc]

Table S3: Significantly expressed genes with function in regulation of cellular and component organization identified by Gene Ontology

| **EntrezID** | **ProbeSetID** | **selected** | **symbol** |
| --- | --- | --- | --- |
| 1050 | 204039_at | 0 | *CEBPA* |
| 10661 | 210504_at | 0 | *KLF1* |
| 1071 | 206210_s_at | 0 | *CETP* |
| 1499 | 1554411_at | 0 | *CTNNB1* |
| 182 | 209097_s_at | 0 | *JAG1* |
| 2038 | 210746_s_at | 0 | *EPB42* |
| 212 | 211560_s_at | 0 | *ALAS2* |
| 2309 | 204131_s_at | 0 | *FOXO3* |
| 3200 | 208604_s_at | 0 | *HOXA3* |
| 3676 | 205884_at | 0 | *ITGA4* |
| 51327 | 219672_at | 0 | *ERAF* |
| 54977 | 217961_at | 0 | *SLC25A38* |
| 55636 | 218829_s_at | 0 | *CHD7* |
| 5925 | 203132_at | 0 | *RB1* |
| 6646 | 221561_at | 0 | *SOAT1* |
| 669 | 203502_at | 0 | *BPGM* |
| 7412 | 203868_s_at | 0 | *VCAM1* |
| 861 | 208129_x_at | 0 | *RUNX1* |
| 8879 | 208381_s_at | 0 | *SGPL1* |
