# Supplementary material for: Frequency and Prognostic Impact of CEBPA Proximal, Distal and Core Promoter Methylation in Normal Karyotype AML: A Study on 623 Cases
Source: PLoS One. 2013 Feb 1;8(2):e54365. doi: 10.1371/journal.pone.0054365 (PMC3562230; doi:10.1371/journal.pone.0054365)
Supplement: Table S4 — Significantly expressed genes with a function for myeloid cell differentiation and hemopoietic development identified by Gene Ontology. (DOC) [file pone.0054365.s006.doc]

Table S4: Significantly expressed genes with a function for myeloid cell differentiation and hemopoietic development identified by Gene Ontology

| **GOBPID** | **Count** | **Size** | **Term** | **p-value** |
| --- | --- | --- | --- | --- |
| GO:0065008 | 54 | 789 | regulation of biological quality | 0,00093937 |
| GO:0051128 | 25 | 265 | regulation of cellular component organization | 0,00148473 |
| GO:0051493 | 11 | 80 | regulation of cytoskeleton organization | 0,01136601 |
| GO:0033043 | 15 | 144 | regulation of organelle organization | 0,01175626 |
| GO:0044087 | 10 | 74 | regulation of cellular component biogenesis | 0,01175626 |
| GO:0042592 | 29 | 401 | homeostatic process | 0,01175626 |
| GO:0006811 | 26 | 344 | ion transport | 0,01175626 |
| GO:0051130 | 11 | 93 | positive regulation of cellular component organization | 0,01623892 |
| GO:0030036 | 15 | 163 | actin cytoskeleton organization | 0,02370808 |
| GO:0030029 | 15 | 167 | actin filament-based process | 0,02734864 |
| GO:0051179 | 92 | 1898 | localization | 0,03256112 |
| GO:0007010 | 21 | 284 | cytoskeleton organization | 0,03256112 |
| GO:0019725 | 18 | 230 | cellular homeostasis | 0,03256112 |
| GO:0006812 | 20 | 268 | Cation transport | 0,03256112 |
| GO:0003013 | 10 | 98 | circulatory system process | 0,0441439 |
| GO:0008015 | 10 | 98 | blood circulation | 0,0441439 |
