# Supplementary material for: Frequency and Prognostic Impact of CEBPA Proximal, Distal and Core Promoter Methylation in Normal Karyotype AML: A Study on 623 Cases
Source: PLoS One. 2013 Feb 1;8(2):e54365. doi: 10.1371/journal.pone.0054365 (PMC3562230; doi:10.1371/journal.pone.0054365)
Supplement: Table S5 — Significantly expressed genes in Gene Ontology. (DOC) [file pone.0054365.s007.doc]

Table S5: Significantly expressed genes in Gene Ontology

| **GOBPID** | **Count** | **Size** | **Term** | **p-value** |
| --- | --- | --- | --- | --- |
| GO:0051128 | 32 | 262 | regulation of cellular component organization | 0,00168679 |
| GO:0003013 | 16 | 92 | circulatory system process | 0,00168679 |
| GO:0008015 | 16 | 92 | blood circulation | 0,00168679 |
| GO:0051188 | 14 | 73 | cofactor biosynthetic process | 0,00168679 |
| GO:0032502 | 126 | 1740 | developmental process | 0,00235825 |
| GO:0065008 | 65 | 765 | regulation of biological quality | 0,0026576 |
| GO:0032879 | 31 | 279 | regulation of localization | 0,0026576 |
| GO:0051179 | 129 | 1842 | localization | 0,00563417 |
| GO:0022603 | 16 | 118 | regulation of anatomical structure morphogenesis | 0,01495767 |
| GO:0022604 | 10 | 57 | regulation of cell morphogenesis | 0,0195869 |
| GO:0051186 | 17 | 135 | cofactor metabolic process | 0,0195869 |
| GO:0051493 | 12 | 79 | regulation of cytoskeleton organization | 0,02057528 |
| GO:0007275 | 94 | 1319 | multicellular organismal development | 0,02057528 |
| GO:0032501 | 116 | 1696 | multicellular organismal process | 0,02057528 |
| GO:0009653 | 45 | 534 | anatomical structure morphogenesis | 0,02140711 |
| GO:0030099 | 14 | 106 | myeloid cell differentiation | 0,02244974 |
| GO:0048870 | 21 | 194 | cell motility | 0,02244974 |
| GO:0016477 | 20 | 181 | cell migration | 0,02244974 |
| GO:0048856 | 81 | 1120 | anatomical structure development | 0,02244974 |
| GO:0048878 | 22 | 209 | chemical homeostasis | 0,02244974 |
| GO:0065007 | 254 | 4247 | biological regulation | 0,02348815 |
| GO:0042592 | 34 | 382 | homeostatic process | 0,02457112 |
| GO:0032989 | 20 | 187 | cellular component morphogenesis | 0,02516049 |
| GO:0033043 | 17 | 148 | regulation of organelle organization | 0,02516049 |
| GO:0030036 | 18 | 162 | actin cytoskeleton organization | 0,02619028 |
| GO:0051049 | 20 | 190 | regulation of transport | 0,028015 |
| GO:0002520 | 22 | 218 | immune system development | 0,02848067 |
| GO:0030029 | 18 | 168 | actin filament-based process | 0,03440216 |
| GO:0048869 | 60 | 805 | cellular developmental process | 0,03440216 |
| GO:0051130 | 12 | 93 | positive regulation of cellular component organization | 0,03494236 |
| GO:0050793 | 53 | 695 | regulation of developmental process | 0,03515719 |
| GO:0044087 | 10 | 71 | regulation of cellular component biogenesis | 0,03717664 |
| GO:0051129 | 11 | 83 | negative regulation of cellular component organization | 0,03765323 |
| GO:0019725 | 21 | 215 | cellular homeostasis | 0,04134159 |
| GO:0050789 | 239 | 4037 | regulation of biological process | 0,04578734 |
| GO:0048534 | 20 | 204 | hemopoietic or lymphoid organ development | 0,04578734 |
| GO:0007010 | 25 | 277 | cytoskeleton organization | 0,04842876 |
