# Supplementary material for: Frequency and Prognostic Impact of CEBPA Proximal, Distal and Core Promoter Methylation in Normal Karyotype AML: A Study on 623 Cases
Source: PLoS One. 2013 Feb 1;8(2):e54365. doi: 10.1371/journal.pone.0054365 (PMC3562230; doi:10.1371/journal.pone.0054365)
Supplement: Table S6 — Overview of CEBPA promoter methylation studies. (DOC) [file pone.0054365.s008.doc]

**Table S6: Overview of *CEBPA* promoter methylation studies**

| **Study** | **Number of patients** | **Karyotype of selected cohort** | **CEBPA promoter region analyzed** | **Analysis method** | **% methylated** |
| --- | --- | --- | --- | --- | --- |
| Hackanson *et al.*, 2008 | 39 | inv(16), t(8;21), t(15;17), (t9;11), complex | distal | BS/  BioCOBRA/  MassARRAY | 51 |
| Lin *et al.*, 2011 | 193 | unselected | distal | Mass Array Anlysis | 14 |
| Chim *et al., 2002* | 70 | not indicated | core | MSP | 2.8 |
| Jost *et al.*, 2008 | 80 | unselected | core | MSP | 12.5 |
| Lu *et al.*, 2010 | 53 | normal | core | MSP | 13.2 |
| Hollink *et al.*, 2011 | 237 | unselected | core | MSP | 1.3 |
| Wouters *et al.*, 2007 | 285 | unselected | proximl region, not further specified | Bisulfide Sequencig | 1.4 |

MSP: methylation specific PCR, BS: Bisulfite Sequencing
